# Vacuolar myopathy with monoclonal gammopathy and stiffness (VAMMGAS)

**DOI:** 10.1111/ene.70026

**Published:** 2025-01-13

**Authors:** Katia Staedler, Yves Allenbach, Emmanuelle Salort‐Campana, Edoardo Malfatti, Aude Rigolet, Shahram Attarian, André Maues de Paula, Sarah Léonard‐Louis, Olivier Benveniste, Tanya Stojkovic

**Affiliations:** ^1^ Groupe Hospitalier Pitié‐Salpêtrière, Institut de Myologie AP‐HP, Sorbonne Université Paris France; ^2^ Groupe Hospitalier Pitié‐Salpêtrière, Département de médecine interne et d'immunologie clinique AP‐HP, Sorbonne Université Paris France; ^3^ INSERM, UMR974 Paris France; ^4^ Centre de Référence des Maladies Neuromusculaires AP‐HM, Hôpital de la Timone Marseille France; ^5^ Referral Center for Neuromuscular Disorders AP‐HP, Henri Mondor University Hospital Créteil France; ^6^ University Paris Est Créteil, INSERM, U955, IMRB Créteil France; ^7^ Département de Neuropathologie AP‐HM, Hôpital de la Timone Marseille France

**Keywords:** elevated CK, monoclonal gammopathy, stiffness, vacuolar myopathy

## Abstract

**Background:**

Monoclonal gammopathy (MG) has been reported in association with numerous neurological disorders but the spectrum of MG‐associated myopathies remains poorly described.

**Objective:**

To report a newly acquired myopathy associated with MG.

**Methods:**

Three adult patients with the same phenotype from two French referral centers were prospectively analyzed. Clinical, electrophysiological, muscle biopsy data, and patients' outcomes under treatment are reported.

**Results:**

The patients, aged 37, 46, and 56 years, presented progressive weakness with subacute worsening and stiffness, in the context of severe weight loss. The weakness mainly involved the proximal limbs and axial muscles. Creatine kinase levels were 1400–2900 IU/L and electromyography revealed a myopathic pattern with spontaneous complex repetitive discharges. Muscle biopsies showed vacuoles containing glycogen and autophagic material along with the presence of sarcolemmal complement membrane attack complex deposits. There was no evidence of a genetic glycogen metabolic disorder. IgGκ monoclonal gammopathy was identified in all cases, without signs of lymphoplasmocytic proliferation. All patients improved with a treatment combining corticosteroids, intravenous immunoglobulins, and immunosuppressants, and two patients recovered walking ability.

**Conclusion and Relevance:**

We report a new muscle disease defined by a vacuolar myopathy characterized by axial and proximal muscle weakness with prominent stiffness and high frequency discharges on electromyography associated with monoclonal gammopathy, defined under the acronym VAMMGAS.

## INTRODUCTION

Monoclonal gammopathy (MG) has been reported in association with several diseases, including kidney diseases and neuromuscular disorders such as peripheral nerve diseases or myopathy [1, 2]. Two muscle diseases linked to gammopathies are recognized: amyloid myopathy (AM), a disorder that can be isolated or secondary to systemic amyloidosis, and sporadic late‐onset nemaline myopathy (SLONM), characterized by severe limb and axial muscle weakness with subacute onset and nemaline bodies on muscle biopsy [3, 4, 5, 6, 7]. In SLONM, the improvement of patients after autologous stem cell transplantation demonstrates the presence of immune‐mediated muscle damage [8]. Here we report a new muscle condition associated with monoclonal gammopathy, enlarging the spectrum of “MG of muscle significance”.

## PATIENTS AND METHODS

### Standard protocol approvals, registrations, and patient consent

Three patients were prospectively identified in two neuromuscular referral centers in France (Pitié‐Salpêtrière Hospital in Paris and Hôpital de la Timone in Marseille). Clinical, electroneuromyography (ENMG), blood tests, and muscle biopsy were analyzed. All patients were examined by at least one of the authors at specialized neuromuscular units and gave their informed consent. The study was conducted in accordance with European Union and French bioethics laws and with the Convention of Helsinki.

### Muscle biopsy

Cryostat sections were stained with hematoxylin and eosin (H&E), modified Gömöri trichrome (mGT), and periodic acid‐Schiff (PAS). HLA‐ABC and C5b9 (W6/32, M0736 and aE11, M 0777 Dako, Glostrup, Denmark), α‐sarcoglycan (Cat # NCL‐L‐a‐SARC, Novocastra, Leica Biosystems), P62/SQSTM1 (Cat # 610833, BD Transduction Laboratories, BD Biosciences) and κ‐light (Cat # F0198, Dako, Agilent) and λ‐light chain (Cat # F0199, Dako, Agilent) antibodies were used for immunohistochemistry. Electron microscopy was performed according to standardized procedures [9].

### Clinical phenotype

#### Patient 1

A 50‐year‐old woman presented with progressive and predominant lower limb and axial weakness starting at age 46. She reported stiffness in abdominal and lower limb muscles. Her neurological examination demonstrated severe axial muscle weakness without a dropped head, and a symmetrical predominant proximal muscle weakness (see Table 1 for detailed MRC score). There was no facial weakness, ptosis, ocular palsy, or macroglossia. There was no myotonia. She had dysphagia, which led to a 10‐kg weight loss in 1 year. Creatine kinase (CK) level was 1600 IU/L. She did not have any myositis‐specific antibodies, but a low‐grade IgG Kappa MG was detected (<1 g/L).

**TABLE 1:** Patients' characteristics.

	Age at onset (years)	Clinical muscular features							Biological features		Response to treatment
		Muscle strength before Tx (MRC)			Muscle strength after Tx (MRC)			Muscle stiffness	MG level before Tx	MG level after Tx	IVIg +/− immunosuppressants
		Axial muscles	Upper limbs R/L	Lower limbs R/L	Axial muscles	Upper limbs R/L	Lower limbs R/L				
Pt 1	46	No DH	D 3/3, TB 3/3, BB 3/3, EC 3/3, FC 3/3, DE 5/5, DF 5/5, DI 4/4	G 3/3, P 3/3, H 4/4, Q 4/4, TS 5/5, TA 5/5	No DH	D 4/4, TB 5/5, BB 5/5, EC 5/5, FC 5/5, DE 5/5, DF 5/5, DI 5/5	G 5/5, P 5/5, H 5/5, Q 5/5, TS 5/5, TA 5/5	Abdominal and lower limb muscles	<1 g/L	<1 g/L	Yes
Pt 2	37	DH	D 3/3, TB 4/4, BB 3/3, EC 4/4, FC 5/5, DE 4/4, DF 5/5, DI 4/4	G 3/3, P 3/3, H 5/5, Q 5/5, TS 5/5, TA 5/5	No DH	D 5/5, TB 5/5, BB 5/5, EC 5/5, FC 5/5, DE 5/5, DF 5/5, DI 5/5	G 5/5, P 3/3, H 5/5, Q 5/5, TS 5/5, TA 5/5	Lower limbs	<1 g/L	U	Yes
Pt 3	56	DH	D 2/2, TB 3/3, BB 3/3, EC 5/5, FC 5/5, DE 5/5, DF 5/5, DI 5/5	P 2/2, H 3/3, Q 3/3, TS 5/5, TA 5/5	No DH	D 4/4, TB 4/4, BB 4/4, EC 5/5, FC 5/5, DE 5/5, DF 5/5, DI 5/5	P 3/3, H 3/3, Q 3/3, TS 5/5, TA 5/5	Lower limbs	11.25 g/L	<1 g/L	Yes

Abbreviations: BB, biceps brachialis; CRD, complex repetitive discharges; D, deltoid; DE, digit extensors; DF, digit flexors; DH, dropped head; DI, dorsal interosseous; EC, extensors carpi; FC, flexors carpi; G, gluteal muscles; H, hamstrings; IVIg, intravenous immunoglobulins; L, left; M, monoclonal gammopathy; MRC, Medical Research Council; P, psoas; Pt, patient; Q, quadriceps; R, right; TA, tibialis anterior; TB, triceps brachialis; TS, triceps surae; Tx, treatment; U, undetectable.

#### Patient 2

A 37‐year‐old woman presented with axial and pelvic muscular weakness, which had developed during the previous 6 months. Her past medical history was unremarkable but her mother had suffered from rheumatoid arthritis and her sister had died from complications related to juvenile dermatomyositis. Three months before the onset of her muscular weakness, she had complained of muscle stiffness in the lower limbs; next, she suffered from muscle weakness and required a wheelchair 6 months later. A 7‐kg weight loss was reported at the same time as the muscular weakness, without dysphagia. Her clinical examination showed severe axial muscle weakness with a dropped head and symmetrical proximal muscle weakness in all four limbs (Table 1). There was no myotonia, cranial nerves examination was normal and there was no macroglossia. CK level was 1400 IU/L. She also presented numerous supra and ventricular extrasystoles but cardiac magnetic resonance imaging (MRI) showed no sign of active myocarditis. She did not harbor any myositis‐specific antibodies but had a low‐grade IgG Kappa MG (<1 g/L).

#### Patient 3

A 60‐year‐old man complained of progressive weakness and stiffness of the lower limbs. At age 63, he walked with an aid. Four years later, he was referred to one of our centers. Examination showed symmetrical proximal weakness in all four limbs (Table 1). This was associated with dysarthria and dysphagia. There was neither macroglossia nor myotonia. He required a wheelchair at age 68 and was referred for a 15‐kg weight loss. At age 71, he had severe proximal weakness in the lower limbs, grade 2–4 on the MRC scale, and proximo‐distal weakness in the upper limbs, graded 3–4, associated with severe axial muscle weakness manifesting with a dropped head. Stiffness of the abdominal and lower limb muscles as well as hypertrophy of the sternocleidomastoid muscles were noted. CK level was 2974 IU/L and protein immunoelectrophoresis showed an IgG Kappa MG (11.25 g/L).

### Nerve conduction studies and electromyography (EMG)

In all three patients, EMG showed a myopathic pattern in all four limbs, characterized by short, small polyphasic motor unit potentials with early recruitment during effort and abundant spontaneous complex repetitive discharges (CRD) in the axial and proximal muscles of all four limbs. There were no myotonic discharges. Nerve conduction velocities were normal in all four limbs.

### Muscle biopsy analysis

**FIGURE 1:**
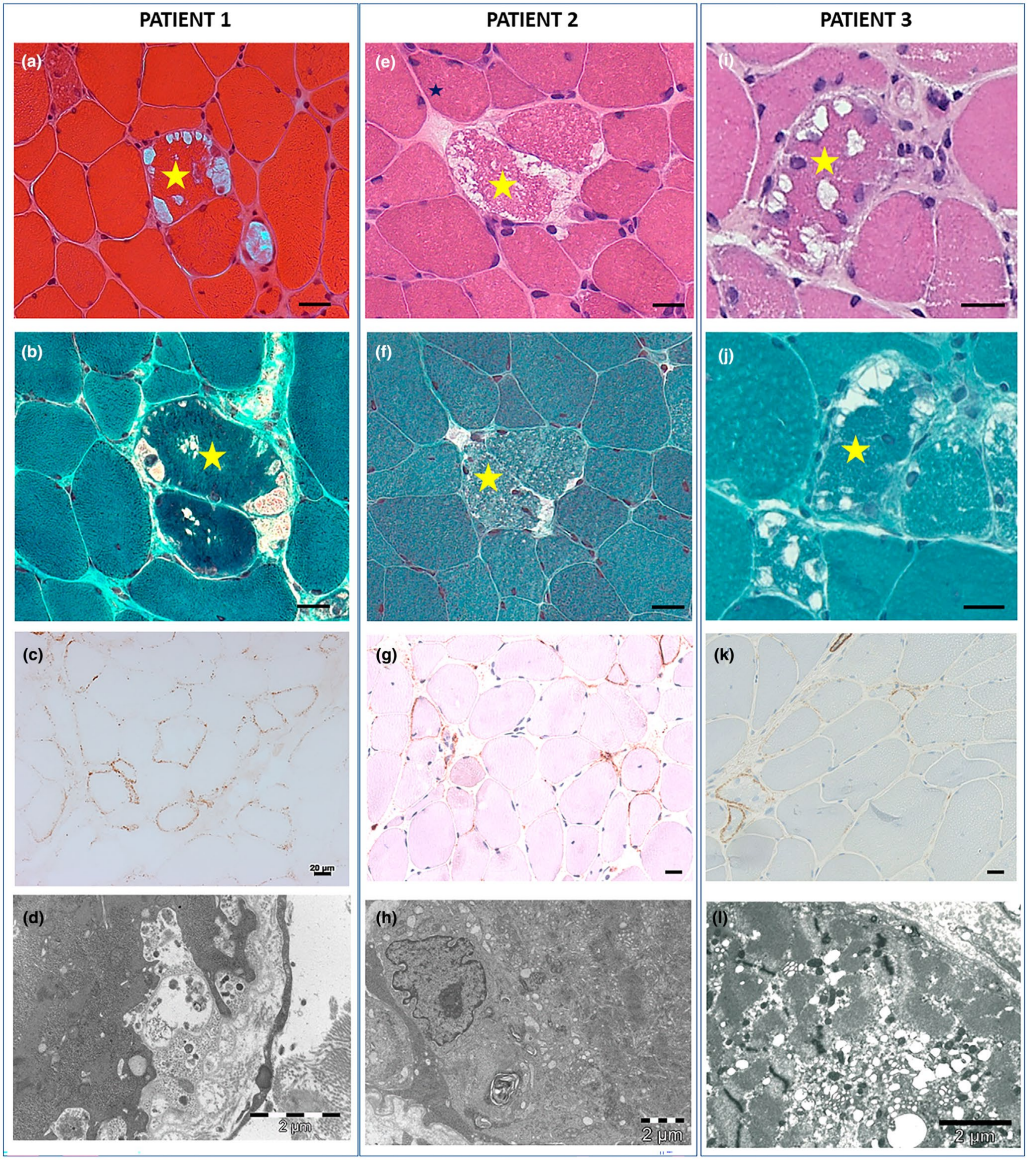
Muscle morphological studies. Deltoid muscle biopsy was performed at ages 53, 37, and 59 in patient 1, 2, and 3, respectively. Cross‐serial sections. Patient 1 (a–d): H&E (a) and mGT (b) showing an abnormal fiber (the same fiber is indicated by a star) harboring multiple subsarcolemmal vacuoles containing some granular/dotted material appearing fuchsinophilic with mGT staining. Marked fiber size/diameter variation is also evident. (c) MAC/C5b9 immunostaining shows the presence of granular complement deposition at the sarcolemma in several fibers. (d) Electron micrograph shows the presence of an abundant subsarcolemmal membrane vacuolization formed by different compartments separated by redundant membrane layers. Autophagic material and cellular debris filled the vacuoles. Scale bars: 20 μm (a–c), 2 μm (d). Patient 2 (e–h): H&E (e) and mGT (f) show a few fibers (one of the fibers is indicated by a star) with multiple subsarcolemmal vacuoles. (g) MAC/C5b9 immunostaining shows the presence of granular complement deposition at the sarcolemma in a few fibers. (h) Electron micrographs showing a completely disrupted muscle cell containing autophagic material with myelinic profiles, cellular debris, and prominent proliferation of reticular structures. Scale bars: 20 μm (e–g), 2 μm (h). Patient 3 (i–l): H&E and mGT (P and S) show a few fibers with multiple subsarcolemmal as well as central vacuoles containing slightly granular material, variations in fiber size and diameter, and rare internalized nuclei. (k) MAC/C5b9 immunostaining shows the presence of granular complement deposition at the sarcolemma in three fibers. (l) The ultrastructural analysis shows multiple sarcoplasmic vacuolization. Scale bars: 20 μm (i–k), 2 μm (l).

Deltoid muscle biopsies (Figure 1) revealed a homogenous histological phenotype showing a vacuolar myopathy with numerous non‐rimmed vacuoles containing amorphous material (Figure 1a–j). Some necrotic/regenerating myofibers were also observed. PAS staining showed variable intermyofibrillar glycogen overload. Congo red staining did not show amyloid deposits within the myofibers or the walls of small arteries. Immunostaining for the detection of protein deficiency associated with limb‐girdle muscular dystrophies was normal; the vacuoles did not show sarcolemma‐like features. There was no P62/SQSTM1‐positive material in patient 1's muscle biopsy. Immunostaining for MHC class I was negative, and C5b9/MAC showed the presence of scattered fibers with granular sarcolemmal deposits (Figure 1c,g,k). Immunofluorescence studies using antibodies against κ‐ and λ‐light chains, performed on patient 1's muscle biopsy, failed to show the presence of κ‐light‐ or λ‐light‐chain‐positive immunoreactive material in non‐vacuolated or vacuolated muscle fibers or muscle vessels. Electron microscopy disclosed the presence of variable glycogen accumulations and abundant vacuolar and autophagic material. Neither rods nor fibrils were observed. Multiplex muscle western blot analysis of sarcolemmal proteins and calpain 3 was normal.

### Genetic analysis and ancillary examinations

The dsosage of enzymes involved in glycogen pathways on muscle homogenate was normal. The acid maltase assay was normal in all patients. Genetic analysis was negative for myotonic dystrophy types 1 and 2 and for muscle sodium, calcium, and chloride channel diseases. In all cases, bone marrow biopsies and whole‐body positron emission tomography (PET), and thoracoabdominal computed tomography (CT) scans were normal. Serum paraneoplastic antibodies were absent.

**FIGURE 2:**
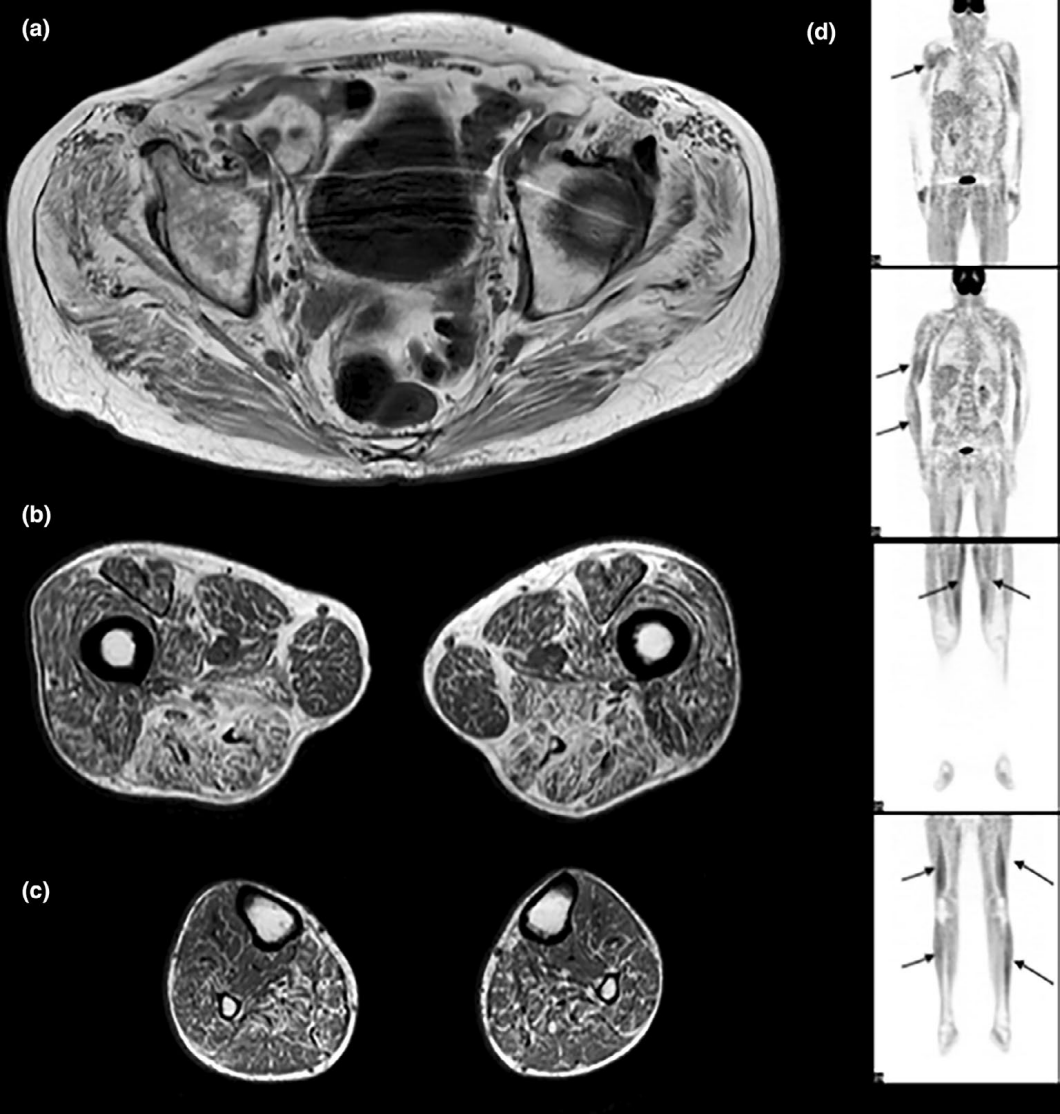
Muscle MRI and ^18^FDG‐PET scan studies in patient 3 at age 68. Muscle MRI of pelvic girdle (a) and thigh (b): fatty degeneration of gluteus, biceps femoris, semimembranosus, semitendinosus, biceps femoris, and adductors sparing gracilis. At the leg level (c), note fatty infiltration of the posterior compartment (soleus, gastrocnemius medialis, gastrocnemius lateralis) sparing tibialis posterior. ^18^FDG‐PET scan (d) shows increased symmetric FDG uptake in both proximal and distal muscles of all four limbs (arrows).

Muscle MRI was normal in patients 1 and 2 at 1 year and 3 months of disease progression, respectively. In patient 3, muscle MRI (Figure 2), performed 9 years after the beginning of the neurological history, showed at pelvic and thigh level a diffuse fatty infiltration of the posterior compartment of the thighs, sparing the gracilis muscle, and at leg level a diffuse fatty infiltration of the posterior compartment, sparing the tibialis posterior. ^18^F‐fluorodeoxyglucose (^18^FDG) PET scan showed a marked symmetric homogenous FDG uptake in the neck, four limbs, and abdominal muscles in patient 3 (Figure 2).

### Treatment and clinical course

#### Patient 1

Due to severe disease evolution causing loss of ambulation an immunosuppressive therapy including high doses of steroids, methotrexate, and plasma exchanges was started 4 years after the first signs, leading to improvement of muscle condition since she was able to walk without any device; moreover, the CK levels were normal. After having stopped plasma exchanges and decreased the prednisolone dosage to below 10 mg/day, the patient relapsed. She then received intravenous immunoglobulins (IVIg) with rituximab, subsequently replaced by revlimid since she experienced relapses when reducing steroid doses while IVIg was maintained. After 2 years of treatment, she was fully ambulant and her muscle strength was normal despite mild deltoid muscle weakness (see Table 1 for details of her MRC score).

#### Patient 2

Six months after the first clinical signs the patient received oral prednisolone with methotrexate and monthly IVIg. Six months later, she was able to walk independently and serum CK levels returned to normal values. When steroid doses were below 20 mg/day and the IVIg doses were decreased, she experienced a relapse. Therefore, methotrexate was switched to azathioprine. After 3 years of treatment, she could walk for 1 hour without help. A residual mild muscle weakness was noted in the iliopsoas muscles (Table 1).

#### Patient 3

At age 70, 10 years after the first symptoms, the patient received high‐dose oral prednisone combined with plasma exchanges and then IVIg infusions. Five months after the first IVIg infusion an improvement was noted as he was able to put his hands on his head and could move from bed to wheelchair unaided. After nine IVIg infusions, he had a moderate proximal weakness in the lower limbs and a mild proximal weakness in the upper limbs (Table 1). He could stand with support but was unable to walk.

In all three patients, after treatment, the CK level was back to normal, and the IgG Kappa MG was either at a very low level (<1 g/L) or even undetectable (Table 1).

## DISCUSSION

These three cases share a clinical phenotype characterized by progressive weakness with subacute worsening of proximal limb and axial muscle weakness, dysphagia, muscular stiffness, and weight loss in a context of elevated CK and IgG Kappa MG. They also share histological findings, with the presence of vacuolar myopathy with glycogen and autophagic material accumulation, complement deposits, without alteration of glycogen metabolic pathways. All patients improved with IVIg in combination with immunosuppressive drugs. This new clinical and histological pattern could correspond to a novel entity, which we have called vacuolar myopathy with monoclonal gammopathy and stiffness (VAMMGAS). It could expand the spectrum of monoclonal gammopathy‐associated myopathies, which includes AM, a pathology that can be isolated if amyloid infiltration affects only the muscles or systemic if muscles are affected with other organs by amyloid infiltration [3, 4], and SLONM. In VAMMGAS there are some arguments for an acquired myopathy: the occurrence of the disease in adult patients, the subacute progression of muscle weakness, and the variable improvement with IVIg and immunosuppressive drugs.

However, there are clear differences between AM, SLONM and VAMMGAS. In VAMMGAS, muscle weakness is constantly associated with stiffness together with high CK levels (>1000 IU/L), a condition not seen in SLONM, where normal or only slightly elevated CK values are found [7, 8, 10]. Normal CK values are also found in systemic AM, while in isolated AM, CK values are usually high [3]. EMG shows a myopathic pattern in VAMMGAS, SLONM, and isolated AM, while in systemic AM it shows a peripheral neuropathy sometimes mixed with a myopathic pattern [3]. In SLONM, fibrillation potentials are frequently found [6, 7, 10] as in isolated or systemic AM^3^, while in VAMMGAS diffuse and abundant CRD are observed. Given the large number of vacuoles observed in our biopsies, the occurrence of these CRD is not surprising since they are also reported in other diseases with vacuoles filled with glycogen, such as Pompe disease [11]. These discharges reflect muscle membrane hyperexcitability and are consecutive to structural sarcoplasmic changes, likely accounting for stiffness reported by the patients [12]. Muscle biopsies in our patients showed numerous sarcolemmal vacuoles, and glycogen and autophagic material accumulation, while SLONM is defined by the presence of nemaline bodies [6, 7, 8, 10]. The mechanism of glycogen accumulation in muscle biopsies is unknown, but this phenomenon has already been demonstrated in inflammatory myopathies, such as dermatomyositis [13]. Soontrapa et al. recently reported a patient combining both glycogen storage disease and polyneuropathy with organomegaly, endocrinopathy, monoclonal gammopathy (IgG kappa and IgA lambda), and skin changes (POEMS) responsive to autologous stem cell transplantation. In contrast to our patients, his muscle biopsy showed a glycogen storage myopathy, polyglucosan bodies, and rimmed vacuoles [14]. An underlying immune‐mediated mechanism in VAMMGAS is supported by the complement deposits. Of note, the presence of C5b9 complement deposition at the membrane is also found in anti‐HMGCR antibodies immune‐mediated necrotizing myopathies, which are variably treatable acquired myopathies [15]. Finally, in SLONM there is an inflammatory component characterized by the occurrence of inflammatory cells, mostly macrophages and cytotoxic T [10], a feature not observed in VAMMGAS. In VAMMGAS there is a good response to IVIg and immunosuppressive treatments, a response infrequently observed in SLONM, which is mainly responsive to high‐dose melphalan followed by autologous peripheral blood stem cell transplantation [8, 10]. The coexistence of rimmed vacuoles and muscle inflammation is also observed in sporadic inclusion body myositis (sIBM), a condition also associated with MG [16]. However, there are striking differences since in VAMMGAS: (i) the vacuoles are not rimmed; (ii) there is prominent autophagic material and glycogen accumulation, without inflammation; (iii) the improvement with immunosuppressants is a feature that distinguishes it from sIBM [17].

We believe the occurrence of an MG in our patients, two of them being under the age of 50 years, is not coincidental, and that there is a link between the two pathologies, given that MGUS in young people is very rare (prevalence <0.3% in people under 40 [18]).

In conclusion, we describe a novel condition characterized by stiffness associated with weakness, abundant complex repetitive discharges, and a vacuolar myopathy, associated with an MG. This condition, VAMMGAS, may be improved by immunosuppressants, and further reports are now needed to confirm this new disease.

## AUTHOR CONTRIBUTIONS

**Katia Staedler:** Conceptualization; writing – original draft; methodology; formal analysis. **Yves Allenbach:** Conceptualization; writing – original draft; formal analysis. **Emmanuelle Salort‐Campana:** Conceptualization; writing – original draft; formal analysis. **Edoardo Malfatti:** Writing – review and editing; formal analysis. **Aude Rigolet:** Formal analysis; writing – review and editing. **Shahram Attarian:** Formal analysis; writing – review and editing. **André Maues de Paula:** Formal analysis; writing – review and editing. **Sarah Léonard‐Louis:** Writing – review and editing; formal analysis. **Olivier Benveniste:** Formal analysis; writing – review and editing. **Tanya Stojkovic:** Writing – original draft; conceptualization; formal analysis; supervision; writing – review and editing; data curation.

## CONFLICT OF INTEREST STATEMENT

None of the authors have any disclosures relevant to the content of the manuscript.

## Data Availability

The data that support the findings of this study are available on request from the corresponding author. The data are not publicly available due to privacy or ethical restrictions.
